# Supplementary material for: The Effect of Age and Comorbidities: Children vs. Adults in Their Response to SARS-CoV-2 Infection
Source: Viruses. 2024 May 17;16(5):801. doi: 10.3390/v16050801 (PMC11126068; doi:10.3390/v16050801)
Supplement: Supplementary file 1 [file viruses-16-00801-s001.zip › viruses-2974580-supplementary.pdf]

Supplementary Table 1. Profile of PCR-positive participants with an IgG titer <15 AU/mL

Identifier[1]	IgG Titer 1 (AU/mL)	Vaccination (Yes/No)	IgG Titer 2 (AU/mL)	Age[2] (years)	COVID-19 symptoms	Any comorbidity (Yes/No)	Comorbidity present
1	3.8	No	N/A	1.5	Mild	Yes	Prematurity; IUGR
2	4.4	No	N/A	1.7	Mild	No	---
3	3.8	No	N/A	12.1	Moderate	Yes	Asthma
4	3.8	No	N/A	16.0	Asymptomatic	Yes	Scleroderma
5	5.2	No	5.4	32.2	Asymptomatic	Yes	HIV
6	9.4	No	N/A	36.1	Asymptomatic	No	---
7	8.4	No	N/A	37.5	Mild	No	---
8	12.4	Yes	166.0	39.8	Mild	Yes	Asthma; Chronic sinusitis; Anemia
9	13.8	No	8.3	40.1	Mild	Yes	HIV
10	12.9	No	N/A	43.2	Mild	Yes	Hepatitis B
11	7.3	No	N/A	46.8	Moderate	Yes	HIV
12	7.9	No	12.3	48.4	Mild	No	---
13	11.8	Yes	400.0	48.7	Mild	Yes	HIV

[1]No two of the above patients belonged to the same family.
[2]Age at first IgG measurement
